# The Effect of Age, Type of Noise, and Cochlear Implants on Adaptive Sentence-in-Noise Task

**DOI:** 10.3390/jcm11195872

**Published:** 2022-10-04

**Authors:** Riki Taitelbaum-Swead, Leah Fostick

**Affiliations:** 1 Department of Communication Disorders, Ariel University, Ariel 4077625, Israel; 2 Medical Division, Meuhedet Health Services, Tel Aviv 6203854, Israel

**Keywords:** HeBio, AzBio, sentence test, adaptive test, SRTn, cochlear implants

## Abstract

Adaptive tests of sentences in noise mimic the challenge of daily listening situations. The aims of the present study were to validate an adaptive version of the HeBio sentence test on normal hearing (NH) adults; to evaluate the effect of age and type of noise on speech reception threshold in noise (SRTn); and to test it on prelingual adults with cochlear implants (CI). In Experiment 1, 45 NH young adults listened to two lists accompanied by four-talker babble noise (4TBN). Experiment 2 presented the sentences amidst 4TBN or speech-shaped noise (SSN) to 80 participants in four age groups. In Experiment 3, 18 CI adult users with prelingual bilateral profound hearing loss performed the test amidst SSN, along with HeBio sentences and monosyllabic words in quiet and forward digits span. The main findings were as follows: SRTn for NH participants was normally distributed and had high test–retest reliability; SRTn was lower among adolescents and young adults than middle-aged and older adults, and were better for SSN than 4TBN; SRTn for CI users was higher and more variant than for NH and correlated with speech perception tests in quiet, digits span, and age at first CI. This suggests that the adaptive HeBio can be implemented in clinical and research settings with various populations.

## 1. Introduction

Mild to profound hearing loss (HL) increased worldwide from 1% in 1985 to 6.1% in 2019 primarily due to age-related demographic changes [1]. As a result, there is an increased need for advanced hearing devices (hearing aids (HA) and cochlear implants (CI)) to assist the greater number of hearing-impaired individuals. Over the last few decades, tremendous advancement has been made in hearing device technology, enabling more individuals to benefit from these devices. Nonetheless, many hearing-impaired individuals experience difficulties listening in noisy, real-life environments. When in a crowded situation, such as a restaurant or a party, they experience a significant increase in listening difficulty and effort and they tend to self-isolate themselves as a result [2]. At the same time, it may still be relatively easy for them to communicate in one-on-one situations. To capture such real-world experiences in the clinic, there is a need for measurements that simulate daily-life situations. Indeed, the most utilized everyday auditory function is speech perception, usually for utterances that include several words (i.e., sentences), and often amidst background noise. In this sense, sentence recognition tests performed in environments of noise have more ecological validity and hence may have a higher correlation with real-world speech understanding [3,4]. However, with the advancements in hearing technology, some current speech recognition tests produce a ceiling effect, especially in quiet conditions [5,6,7,8,9]. Additionally, sentences in quiet do not reflect the daily challenge of understanding speech accompanied by noise. Thus, there is a need for new tests for speech perception, especially in noisy conditions.

When considering hearing tests, especially speech recognition with accompanying noise, several issues should be considered: (1) testing methods—fixed versus adaptive signal-to-noise ratio (SNR); (2) scoring; (3) types of noise; (4) and populations in relation to age and type of impairment.

### 1.1. Testing Method

One key challenge with speech perception tests with accompanying noise involves setting appropriate SNR levels to avoid ceiling and floor effects. With a fixed SNR method, each individual is tested in several SNR conditions, resulting in an accuracy rate for each SNR. This method allows intensive assessment, with an equal number of stimuli for each SNR. However, such testing is lengthy to conduct, and as such, unfeasible in clinical settings for many populations [10,11]. Adaptive tests, on the other hand, allow the use of background noise with changing levels of SNRs according to participant performance. For both methods, the speech receptive threshold (SRT) is the SNR required for a 50% intelligibility score. However, the fixed SNR method assesses the SRT from multiple fixed SNRs, while the adaptive SNR method estimates it directly during participant performance [12]. As the direct assessment of the adaptive SNR method allows for a much shorter testing time, this procedure was evaluated in the current study.

### 1.2. Scoring

Another important issue when considering new speech perception tests is the scoring method, namely, how it is determined whether sentences were perceived correctly, as this measure defines the SRT. There are various scoring methodologies: scoring a whole sentence as one unit (a score of ‘correct’ when all words in the sentence were perceived correctly), scoring each word separately (providing a numeric score reflecting the percentage of words perceived correctly in the sentence), scoring according to the function of the word in the sentence (e.g., lower scores for function words than for nouns, adverbs, and verbs), or scoring predefined keywords. Scoring each word separately will reflect a listener’s partial perception (perceiving some words in the sentence) while scoring the entire sentence as one unit will not [13]. Moreover, findings suggest that scoring fewer units per sentence (such as scoring the entire sentence as a single unit) may result in a higher standard deviation in repeated measurements because missing one word in a sentence will result in its being scored as ‘incorrect’ [14]. The scoring debate will be tested in the current study.

### 1.3. Type of Noise

When testing speech perception under noise conditions, there is a question regarding the type of noise one should use. There are two main types of noise, providing either energetic or informational masking. Energetic masking interferes at the peripheral level by activating the basilar membrane at locations similar to those stimulated by the energy in the speech wave. Informational masking affects speech processing also at central and cognitive levels by presenting competing similar stimuli to the target speech [15]. Indeed, there is a critical role played by central factors such as attention, memory, and linguistic processing. Steady-state speech-shaped noise (SSN), frequently utilized in laboratory and clinical testing, exerts energetic masking on target speech. However, multi-talker babble noise comprising few speakers also exerts informational masking that may cause misattribution of noise components for target speech and increase competition for attention, thus increasing both cognitive load and linguistic interference. As a result, adaptation to SSN is easier because it is a stable noise, while multi-talker babble noise has dips and changes over time, in addition to the challenge of separating target speech from background noise [16]. In the current study, we used both SSN and four-talker babble noise (4TBN) to represent a range of challenging listening demands within various common environment situations: filtering out the multi-talker babble noise of restaurants or a nearby competing conversation and dealing with environments providing steady-state noise, such as air conditioning.

### 1.4. Population

Speech perception in noise is affected by age and changes across the lifespan, evolving from infancy to adulthood [17]. It deteriorates later in life due to a decrease in peripheral hearing thresholds, as well as central systems and cognitive function [15,18]. School-age children and older adults tend to perform more poorly than young adults on speech recognition in noise tasks, especially when stimuli are accompanied by babble noise [19,20,21]. However, their difficulty may be related to different mechanisms. This has yet to be proven, since there are only a few studies that assessed speech perception in noise across the lifespan. The present study aimed to contribute to filling this knowledge gap by testing speech perception in noise across life span.

In addition to age-related changes in hearing ability, CI also affects speech perception. CI devices have limitations in transmitting full spectral information (poor “bottom-up”) which leads to difficulties recognizing degraded signals, especially under adverse listening conditions. Thus, when an auditory signal is degraded, the CI user has to rely on cognitive abilities (e.g., working memory (WM)) and linguistic abilities (“top-down” processes) to match the auditory input with its long-term stored representations [22]. However, prelingual CI users have poorer verbal WM than normal hearing (NH) peers, as well as poorer linguistic skills [23], so their speech perception may be particularly disadvantaged. Only a few studies assessed speech in noise among prelingual adolescent and young adult CI users. Most of those studies evaluated sentence identification in noise using a fixed SNR condition [24,25] with wide variability in the testing conditions (sentences and noise types, SNRs, etc.); not surprisingly, the reported results also varied widely. The limited number of studies that actually evaluated prelingual adolescent and young adult CI users using adaptive procedures, however, found significantly higher thresholds for CI users compared to their NH peers [26]. Further, some background factors, such as residual hearing, and ages at onset of deafness and implantation, were found to be related to speech in noise perception among prelingual CI users [27,28]. The present study also considered the issue of CI use by comparing their performance on the adaptive HeBio to their NH peers.

### 1.5. The Current Study

The increasing need for new, sufficiently adequate speech perception tests has led to the development of a new sentence test, the AzBio [29]. This tool includes many sentence lists (33 in Hebrew, 32 in English, 30 in French, and 42 in Spanish) of similar difficulty. The sentences are designed to be meaningful, but not expected (for example, “Your hair was colored green”), that are spoken by multiple talkers. Accordingly, they provide a good estimation of performance in daily listening conditions (English [29]; Hebrew [30]; French [31]; Spanish [32]).

In a previous study, we adapted and validated the English AzBio sentence test to Hebrew [30]. This study showed that the HeBio had 33 lists of similar intelligibility scores among both NH and CI groups. The HeBio provides an unbiased evaluation tool for those who achieved ceiling effects and high exposure to traditional sentence list tests and can also be administered to HA wearers and older populations. The current study was carried out in an attempt to further improve the HeBio test by considering the issues of methods, scoring, noise, and populations. We implemented the HeBio as an adaptive procedure (Adaptive HeBio), by testing its reliability and the most suitable scoring criteria (Experiment 1), its SRT with different types of background noises and among different age groups (Experiment 2). In addition, we tested its applicability as an adaptive tool for CI users (Experiment 3).

## 2. Experiment 1: Scoring and Reliability

The goal of Experiment 1 was to examine which of the scoring criteria (“All Words” or “At Least Four Words”) would provide SRTn similar to those presented in the literature. Subsequently, we aimed to examine the test–retest reliability of the Adaptive HeBio in measuring SRTn.

### 2.1. Materials and Methods

#### 2.1.1. Participants

Forty-five participants (17 men, 28 women), aged 20–36 years (mean = 24.5 years, SD = 3.06), were recruited for the purpose of developing the Adaptive HeBio. The participants were all native Hebrew speakers, and none had been diagnosed with a learning disability or attention deficit hyperactivity disorder (ADHD). All participants were screened for hearing ability using an Interacoustics-AD629 audiometer and were found to have a hearing level for pure tone signals of less than 20 dB HL for 0.5, 1, 2, and 4 kHz. All participants received an oral explanation of the research and provided signed informed consent.

#### 2.1.2. Tools and Materials

The HeBio includes 33 sentence lists that were aimed to reflect examples of daily adult discourse and to be meaningful yet not predictable (for more details, see [30]). Each list includes 20 sentences, recorded by four talkers (two men, two women, five sentences each). The sentences were constructed in Hebrew in the same method as the English version [29]. They were composed without restrictions on complexity, vocabulary, or phonemic content but did not include names of people, places, or objects. The sentences contain 2–12 words (M = 6.12, SD = 1.70), with one sentence containing two words, one sentence containing 12 words, and six-word sentences being the most prevalent. In the current experiment, sentences were presented amidst 4TBN. The 4TBN included two male and two female talkers. Each talker recorded a different text in Hebrew and the recordings were normalized to have the same root mean square (RMS) amplitudes and were combined into a single recording of four-talker babble noise with a frequency range of 0.5–5 kHz.

#### 2.1.3. Software and Equipment

Auditory stimuli (i.e., sentences) were delivered with a unique Advanced Bionics (AB) adaptive software from a Lenovo (Quarry Bay, Hong Kong) laptop computer via a Genelec (Älvsjö, Sweden) 810A loudspeaker and MAYA 44 (Leonberg, Germany) external sound card. The intensity was calibrated to 65 dBSPL using a sound level meter with a calibration tone of 1000 Hz. Hearing screening was performed using an Interacoustics (Middelfart, Denmark) AD629 audiometer.

#### 2.1.4. Adaptive Procedure

During testing, noise intensity was varied adaptively using a one-down/one-up procedure to target the SNR at which 50% of the speech was identified correctly (i.e., the SRTn). The initial SNR was +10 dB with a step size of 5 dB. When participants did not repeat a sentence correctly (according to the criterion), the noise was decreased/increased in 2 dB steps. The steps of the intensity remained 2 dB until the end of the list. Estimated SRTn thresholds for each list were calculated according to the mean SNR of the last ten sentences in each list.

The intensity of the speech was kept constant at a level of 65 dBSPL which was chosen so that the utterances were clearly audible for all participants.

#### 2.1.5. Procedure

Participants sat in a quiet room at a distance of one meter from a loudspeaker. The sentence lists included 20 sentences, and each list was chosen randomly from among the full set of 33 lists. Each participant received four lists, randomly chosen. Two lists were scored as “correct” in the condition when all words were repeated correctly (“All Words” criterion), and two of which were scored as “correct” in the condition when at least four words of a sentence were repeated correctly (“At Least Four Words” criterion). For sentences shorter than four words, all words needed to be repeated correctly. Examination of test–retest reliability was performed on the two lists presented adaptively with the “At Least Four Words” criterion. Only binary correct/incorrect scores were considered (and not a numeric score of number of words, as was used in Taitelbaum-Swead et al., 2022) in order to define the SNR for the sentence in the next trial.

### 2.2. Results

#### 2.2.1. Scoring

Scoring two sentence lists according to the “All Words” criterion resulted in a mean SRTn of 2.06 dB and 1.52 dB (SD = 1.05 and 1.22), while scoring two sentence lists according to the “At Least Four Words” criterion resulted in a mean SRTn of −0.22 dB and −0.03 dB (SD = 1.13 and 0.94) for the first and second lists, respectively. A two-way repeated-measures ANOVA was conducted on SRTn scores with list number (first, second) and scoring criteria (All Words, At Least Four Words) as within-subject variables. A significant main effect was found for scoring criteria (F(1,24) = 109.73, *p* < 0.001, partial η^2^ = 0.821), confirming that the scoring criterion of “At Least Four Words” provided lower SRTn than the criterion of “All Words”. No main effect was found for list number (F(1,24) = 0.857, *p* = 0.364, partial η^2^ = 0.034) nor was a Method × List interaction found (F(1,24) = 2.969, *p* = 0.098, partial η^2^ = 0.110).

#### 2.2.2. Test–Retest Reliability and Psychometric Data

**Figure 1:**
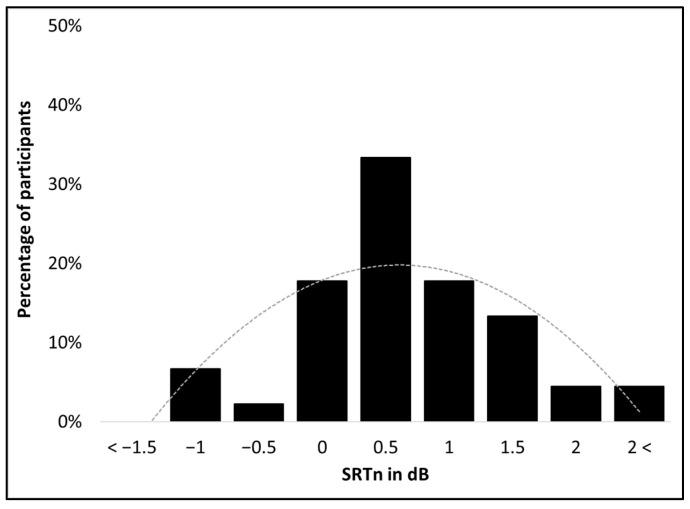
Distribution of SRTn values for the Adaptive HeBio.

Test–retest reliability was performed on lists scored with the “At Least Four Words” criterion and had lower SRTn values. Thresholds for all 45 participants across the two lists were −1.80 to 1.80 dB, with a mean threshold of −0.02 dB (SD = 0.79). The distribution was not significantly different from normal (Shapiro–Wilk (45) = 0.986, *p* = 0.843; Kolmogorov–Smirnov (45) = 0.092, *p* = 0.200), and was neither skewed (Skewness = −0.024, SE = 0.354) nor kurtotic (Kurtosis = 0.132, SE = 0.695) (see Figure 1). Test–retest reliability conducted between the first and second lists showed no significant difference between them (t(44) = 0.057, *p* = 0.876).

**Figure 2:**
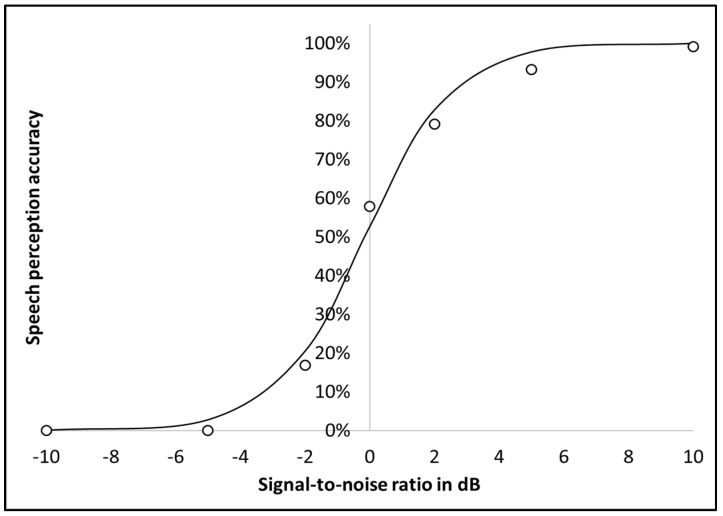
Psychometric function of SNR thresholds of the Adaptive HeBio.

The psychometric function for the mean of the two lists was calculated using a logistic curve and is presented in Figure 2. It provided an SRTn of −0.15 with a slope of 16%. The psychometric function was also calculated separately for each list. The slope was 14% for the first list and 18% for the second. Thus, there was no significant difference in the slopes of the psychometric function between the first and second lists (t(36) = 0.61, *p* = 0.55).

### 2.3. Discussion

The Adaptive HeBio, which called for the identification of sentences amidst four-talker babble noise, provided a mean SRTn of approximately 0 dB for NH young adults when scoring according to comprehension of most of each sentence (“At Least Four Words” criterion) and a slope of 16%. To perform the HeBio in the adaptive procedure, there is a need for a binary correct/incorrect scoring upon which the SNR in the next sentence is decided. The “At Least Four Words” criterion” provided SRTn and slope that are consistent with reported data on the same test material (sentences) and noise condition (4TBN) [33,34,35,36]. Importantly, the test was found to be reliable, with no significant differences in thresholds and slopes between the test and retest lists (20 sentences each), suggesting no learning effect or fatigue from list 1 to list 2.

The slope in the current study was relatively steep, which indicates that a small change in SNR results in large performance differences. The slope of 16% in the Adaptive HeBio is equivalent to those reported for tests such as the new Everyday Conversational Sentences in Noise test (16.3%/dB for females and 18%/dB for males [34]) or for the Matrix test in some languages (German: 17.1%/dB [36]; Polish: 17%/dB [35]). However, all of these reported slopes were steeper than those reported for the HINT and BKB sentence tests (10.3%/dB [37] and 10%/dB [38], respectively). Some factors may lead to variability in the slope steepness, such as context effects, linguistic complexity, and type of masker [39]. Although a steep slope is important for test sensitivity, it is very important that psychometric functions reflect the experience of the listener in the noisy conditions of daily life. While the relationship between the psychometric functions of any particular speech test and real-world communication performance is not entirely clear, nevertheless, it is better to utilize a test that appears to reflect real-world performance.

## 3. Experiment 2: Age and Noise

The goal of Experiment 2 was to test the Adaptive HeBio’s sensitivity to different age groups and types of noise, along with test–retest reliability among these different age and noise conditions.

### 3.1. Materials and Methods

#### 3.1.1. Participants

Eighty participants were recruited across four age groups: 20 adolescents (15 females, aged 12–15 years, mean 13.45 years, SD = 1.10), 20 young adults (13 females, aged 21–30 years, mean 25.15 years, SD = 2.43), 20 middle-aged adults (12 females, aged 40–56 years, mean 46.80 years, SD = 4.53), and 20 older adults (11 females, aged 65–75 years, mean 68.70 years, SD = 3.60). All participants were native Hebrew speakers and were screened for age-normal hearing at 500, 1000, 2000, and 4000 Hz [40]. Hearing threshold criteria were less than or equal to 20 dB in the young groups and less than or equal to 30 dB in the group of older adults. Older adults were screened for cognitive decline using the Montreal Cognitive Assessment (MoCA) test; all participants received a score of 26 or higher (out of 30).

#### 3.1.2. Adaptive Threshold Estimation

The procedure resembled that of Experiment 1. Each participant was presented with two lists for each noise condition, and scoring was conducted according to the “At Least Four Words” criterion. There were two noise conditions: 4TBN as described in Experiment 1 and speech-shaped noise (SSN). The SSN was a steady-state narrow-band noise that matched the existing speech signals and was created using the Fast Fourier Transform of all speech files. The attenuation rate of the SSN was 12 dB/octave from 1000 Hz. Both noises had similar frequency ranges and long-term-average-speech-spectra as the sentences, and were normalized to have similar RMS amplitudes as the sentences.

### 3.2. Results

**Figure 3:**
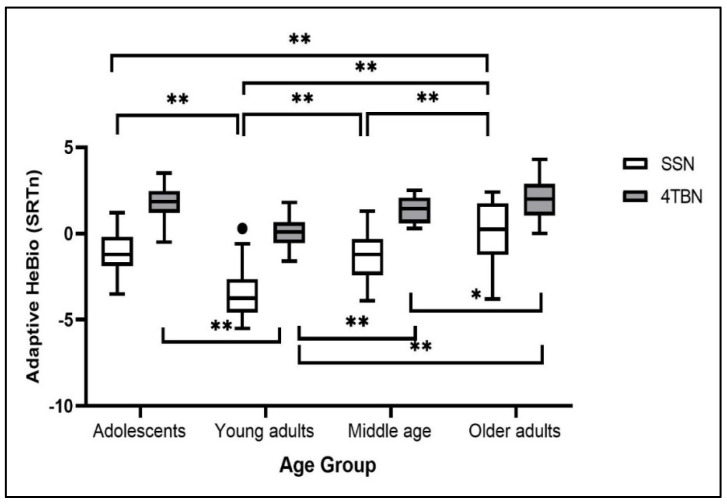
Box-and-whisker plot of SRTn values on the Adaptive HeBio for two types of noise: Speech Shaped Noise (SSN) and four-talker babble noise (4TBN) across four age groups. Dot represents observation outside the interquartile range. * *p* < 0.05; ** *p* < 0.01 LSD test.

Figure 3 presents the SRTn range in each age group and the two noises (4TBN and SSN). A three-way mixed-model repeated-measures ANOVA was carried out on SRTn data with List (first, second) and Noise (4TBN, SSN) as within-subjects variables and age Group as a between-subjects variable. Power analysis showed a power of 90% for medium effect size, based on a sample size of 80 participants (G*Power 3.1.9.4). There were main effects for Noise (F(1,76) = 283.324, *p* < 0.001, partial η^2^ = 0.788) and Group (F(3,76) = 25.350, *p* < 0.001, partial η^2^ = 0.500), but no main effect for List (F(1,76) = 0.060, *p* = 0.807, partial η^2^ = 0.001). There was a Noise × Group interaction (F(3,76) = 4.553, *p* = 0.005, partial η^2^ = 0.152), but the interactions with List were not significant (List × Noise: F(1,76) = 0.252, *p* = 0.617, partial η^2^ = 0.003; List × Group: F(3,76) = 0.315, *p* = 0.814, partial η^2^ = 0.012; List × Noise × Age: F(3,76) = 0.658, *p* = 0.580, partial η^2^ = 0.025). SSN resulted in an overall lower mean SRTn (mean = −1.40, SD = 0.75) than 4TBN (mean = 1.34, SD = 0.47). A post hoc one-way ANOVA showed a main effect for age in both SSN (F(3,76) = 19.013, *p* < 0.001, partial η^2^ = 0.429) and 4TBN (F(3,76) = 16.573, *p* < 0.001, partial η^2^ = 0.395). However, post hoc Least Significant Difference (LSD) showed a small difference between the noises in the differences between age groups. In both noises, there were differences between all groups, except that the older adults were not different from adolescents. However, in 4TBN, there was also no difference between adolescents and the middle-aged (see Figure 3).

### 3.3. Discussion

Following Experiment 1’s demonstration of the reliability of the Adaptive HeBio, the aim of Experiment 2 was to test its sensitivity to age and to two types of noise (SSN, 4TBN) representing energetic masking and informational masking. Regardless of the type of noise, Adaptive HeBio thresholds were sensitive to increases in age, evidencing a decrease in SRTn from adolescents to young adults, and an increase in SRTn from middle-aged to older adults. These findings are in line with other studies that evaluated the effect of age on speech in noise tasks across the lifespan (children and adults [41]; young, middle-aged and older adults [19]; children, young and older adults [42]).

The Adaptive HeBio was also sensitive to the type of noise, with better SRTn values (more negative) for energetic noise (SSN) and worse SRTn values for energetic and informational (4TBN). Other studies also found better STRn values for energetic noise compared to multitalker babble noise [43]. These differences between the two noises may be attributed to the fact that informational masking affects hearing at the central and cognitive levels as well as the peripheral level. The challenge the 4TBN poses to the listeners might be the reason for the noise × group interaction in which adolescents performed at a similar level as older adults. Thus, this type of noise appears to compete for the same attentional resource as speech stimuli. Moreover, test–retest reliability again showed no fatigue or learning effects, which re-confirms the high reliability of the Adaptive HeBio, even among different age groups and amidst different background noise.

## 4. Experiment 3: Cochlear Implant Users

The goal of Experiment 3 was to evaluate the implementation of the Adaptive HeBio among congenitally or early deaf participants using cochlear implants. Its criterion validity was determined in relation to other speech perception measures, and age at first CI. Additionally, a possible association between performance on the Adaptive HeBio and the basic cognitive ability of working memory was measured.

### 4.1. Materials and Methods

#### 4.1.1. Participants

Eighteen CI users (12 women) with a mean age of 21 years (SD = 6) participated in the study. All were either congenitally deaf or became deaf in their first two years of life. Of them, 15 participants had two CIs. The mean age of the first (or only) cochlear implantation was 4.44 years (SD = 4.8), and the mean age of the second implantation was 11.2 years (SD = 6.8). All CI users were native Hebrew speakers recruited for the study via Israeli associations for the hearing impaired. Seventeen participants were using the Cochlear device, and one had an Advanced Bionics device. All participants had studied in a mainstream education setting (for detailed data on the participants, see Table 1). They or their parents provided signed informed consent to participate in the study and received monetary compensation for their participation time.

#### 4.1.2. Speech Perception Tests

##### Adaptive HeBio Sentence Test

The form of the Adaptive HeBio resembled that of Experiments 1 and 2: each participant was presented with one list of sentences accompanied by SSN, and scoring followed the “At Least Four Words” criterion.

##### HeBio Sentence Test (In Quiet)

The test included 33 sentence lists. Each list included 20 sentences, of which five sentences from each of four talkers (2 men, 2 women) were recorded. Since this test was not adaptive, scoring was expressed as the percentage of correct words repeated by the subject. Listeners were instructed to repeat each sentence and encouraged to guess when unsure [30].

##### CVC Monosyllabic Words

Fifteen lists of 10 meaningful, one-syllable, consonant–vowel–consonant phonetically balanced Hebrew words were composed according to the Arthur Boothroyd (AB) words test [44,45] (i.e., in each list, every consonant appears once, and every vowel appears twice). For each participant, two lists of 10 words each were presented. Participants were instructed to repeat each word they heard and to guess if they were unsure. Results were manually recorded by the experimenter and expressed as the percent of correct words.

#### 4.1.3. Cognitive Test

##### Auditory Forward Digit Span Test

Sets of random digits are presented aloud at a rate of one per second, with instructions to report them back verbatim in the order in which they are heard. The shortest set contains two digits and increases in the number of digits progressively until the individual is no longer able to recall all of the digits accurately and in the correct order. Participants receive two sets of each length, and the individual’s “span” is recorded as the maximum number of digits at which at least one of the two sets is accurately recalled [46].

#### 4.1.4. Design and Procedure

CI users sat in a quiet room one meter from a loudspeaker. Recordings of the auditory stimuli (i.e., sentences and words) were delivered from a Lenovo laptop computer via a Genelec 810A loudspeaker and MAYA 44 external sound card. The intensity was calibrated to 65 dB SPL using a sound level meter with a calibration tone of 1000 Hz. The Adaptive HeBio accompanied by SSN was administered as described in Experiment 2. Bilateral CI users (*n* = 15) were tested wearing both CIs, whereas those with bilateral deafness using only one CI (*n* = 3) were tested with their only implant device. Participants were tested first with the HeBio in quiet and then with the Adaptive HeBio, CVC word recognition test, and auditory digit span (conducted in monitored live voice), in random order. Two participants received accuracy scores <40% on the HeBio in quiet and therefore were not tested with the Adaptive HeBio. Additional two CI users were excluded from the result analysis because they received a SRTn threshold of more than 20 dB SNR.

### 4.2. Results

#### 4.2.1. CIs vs. NHs

**Figure 4:**
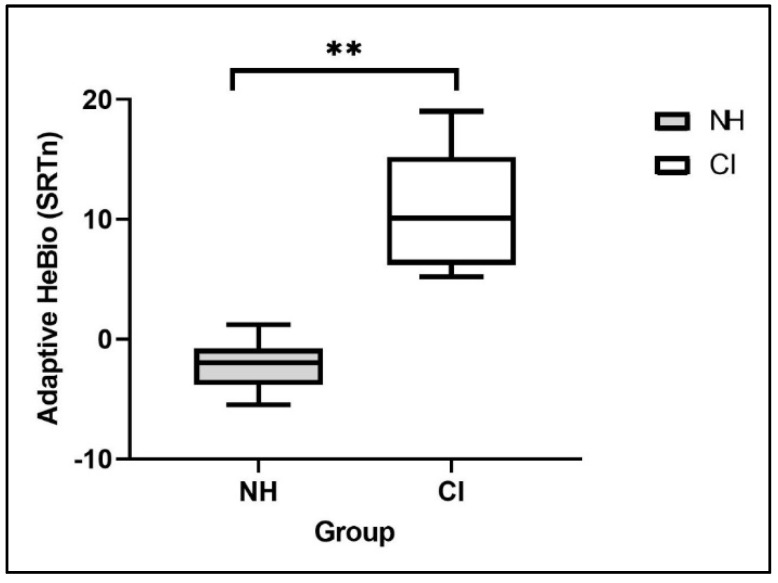
Box-and-whisker plot of the SRTn values on the Adaptive HeBio by group: prelingual Cochlear Implants (CI) users and Normal Hearing (NH) young adults. ** *p* < 0.01.

Figure 4 presents the range of SRTn values during the Adaptive HeBio testing for the CI group as compared to an NH group (young adults tested in Experiment 2). The mean SRTn was 10.9 dB for the CI users compared to −2.2 dB for the NH young adults. Independent sample *t*-testing showed this difference to be significant (t(52) = 14.9, *p* < 0.0001). Large between-subject variability can be observed in the CI group, whose SRTn values ranged from 5.2 dB to 19 dB (a range of 13.8 dB SNR), compared to values of −5.5 dB to 1.2 dB (a range of 6.7 dB SNR) in the NH group.

#### 4.2.2. Validity for CIs

**Figure 5:**
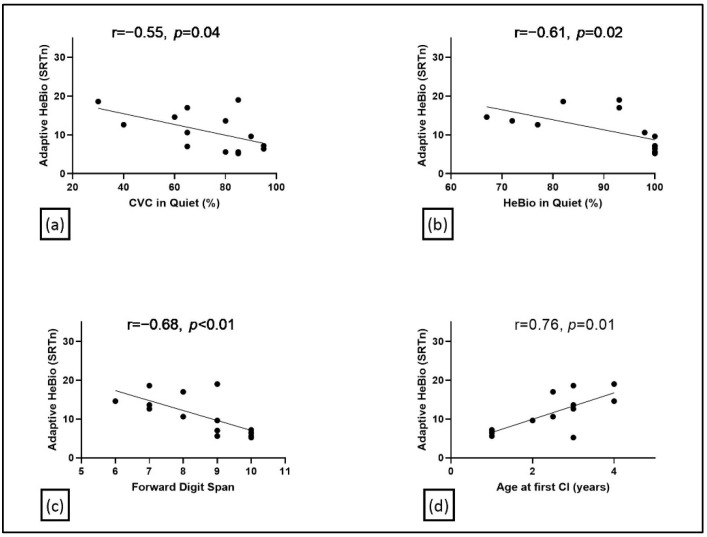
Individual results of Speech Receptive Thresholds in noise (SRTn) values users on the Adaptive HeBio in Speech-Shaped Noise (SSN) for Cochlear Implants (CI) users as a function of (**a**) percentage of correct words on Consonant-Vowel-Consonant (CVC)word recognition test in quiet; (**b**) percentage of correct words on the HeBio sentence test in quiet; (**c**) results on the forward digit span and (**d**) age at 1st CI.

Figure 5 presents SRTn values on the Adaptive HeBio in SSN as a function of the percent accuracy on the CVC word recognition test in quiet (Figure 5a) and on the HeBio in quiet (Figure 5b). Pearson coefficient correlation testing revealed a significant negative association between the percentage accuracy on the Adaptive HeBio SRTn and those on the CVC in quiet and on the HeBio in quiet. These results indicate that CI users with lower SRTn values (thus, better performance) achieved higher scores on the CVC and HeBio in quiet. CVC words accuracy explained 30% of the variance in Adaptive HeBio accuracy while the HeBio in quiet explained 37%.

Figure 5d shows the age at first (or only) CI as a function of SRTn. Pearson coefficient correlation testing revealed a significant high negative association between age at first CI implantation and the SRTn on the Adaptive HeBio, with this factor explaining 58% of the variance in the Adaptive HeBio SRTn.

#### 4.2.3. Adaptive HeBio and Cognition

Individual auditory forward digit span scores as a function of the SRTn on the Adaptive HeBio in SSN are shown in Figure 5c. Pearson correlation testing revealed a significant negative correlation between these variables, suggesting better sentence intelligibility with better working memory scores. This cognitive factor explained 47% of the variance on the Adaptive HeBio SRTn.

### 4.3. Discussion

The purpose of Experiment 3 was to examine whether the adaptive version of the HeBio could be administered successfully to prelingual CI users, to test its validity among this group, and to explore its possible relationship to working memory. The findings of CI users administered the Adaptive HeBio sentence test accompanied by SSN showed wide variability in their SRTn (5.2–19 dB). However, their thresholds on this version of the test were highly correlated with validated speech perception tests in quiet (CVC, HeBio) as well as with cognitive measures (auditory forward digit span). In addition, adult CI users who were implanted at a younger age evidenced more negative scores (thus better SRTn values) on the adaptive version, in accordance with our expectations.

The original English AzBio sentence test is routinely administered in quiet or in noise with a fixed SNR. In the clinical settings, one AzBio list is presented in quiet and if the speech perception accuracy exceeds 60% it is then presented in a +10 dB SNR. If the result with this SNR exceeds 60% accuracy, it is then also presented in a +5 dB SNR [47]. In a relatively recent study by Brant et al. [9], CI users who were tested using an SNR of +10 dB demonstrated a mean score of approximately 50%, while an SNR of +5 dB produced a score of just over 30%. In comparison, in our data, half of CI users achieved SRTn values better than +10 dB. This shows a similarity between our findings measured using the adaptive procedure and those presented in the literature (tested at a fixed SNR), although the participants in the present study are different from those presented in other studies (postlingually deaf adults). Moreover, the adaptive procedure is shorter to conduct and has minimal limitations of floor and ceiling effects.

When reviewing other adaptive sentence tests used to evaluate CI users, a variety of findings were observed. In recent studies utilizing the Matrix sentence test, Italian-speaking high performer CI users were found to demonstrate a mean SRTn of 4.15 dB, while Hebrew-speaking prelingual CI users demonstrated a mean SRTn of 1.3 dB amidst a wide range of −3.7 to +14 dB [3,26]. The current study showed a mean SRTn of 10.9 dB with a wide range of 5.2–19 dB. This difference in SRTn may be explained by various differences between the tests: one such difference is that Hebrew Matrix scores were based on the third and fourth lists administered, while the Adaptive HeBio was based on the first list administered (20 sentences). Thus, lower thresholds on the Matrix could reflect a practice effect. Second, as the HeBio involves four different speakers for each presented list, the listener needs to tune in to different speakers’ acoustic characteristics (“speaker normalization”) which requires more mental resources and makes the perception of the sentences more difficult. Third, although the HeBio sentences are logical, their content has low predictability. All these factors suggest the HeBio might be more difficult than the Matrix, contributing to the higher SRTn values.

Our finding of a high correlation between the SRTn values for the Adaptive HeBio and the results of HeBio and CVC monosyllabic word recognition tests in quiet are in line with other studies [9,33]. This finding supports the supposition that a precondition for good speech perception in noise is good speech perception in quiet. Noteworthy is the fact that high scores on monosyllabic word recognition indicate good speech signal transmission by the CI because these words have less linguistic redundancy, and one must perceive them based mainly on spectro-temporal information transmitted by the CI.

Our findings also showed a positive relationship between short-term memory (based on forward digit span) and speech-in-noise performance in the Adaptive HeBio sentence test. These results may reflect the high cognitive resources involved in the restoration of degraded auditory speech stimuli provided by the CI. Accordingly, the “ease of language understanding” model, which focuses on the role of cognitive processes in speech perception [22,48], indicates that when the auditory signal is degraded, it is difficult to match auditory input with its long-term stored representations. In these conditions, listeners make larger use of working memory to retain the input and resolve this mismatch. Therefore, CI users with larger working memory capacities may perform better in speech perception in difficult listening conditions [22,48] as our findings have shown. This could also be the case with other populations, and future studies may also test this relationship in NH listeners in different age groups, as well as other hearing-impaired listeners.

Age at implantation is also an important factor that affects the capabilities of CI users. Our findings suggest that prelingual adolescents and young adults with CIs who were implanted with their first one or two implants at a younger age performed better in the sentence-in-noise task in the current study. Indeed, six CI users (out of 18) who were implanted before two years of age had SRTn values better (lower) than 10 dB, and two CI users who were implanted after the age of 6 years, had the worst (highest) SRTn values. It should be noted that the thresholds of the CI users who were implanted at an early age were higher than those of NH participants, demonstrating that some individuals with CIs could achieve SRTn values similar to the lowest scoring NH participants. Notwithstanding, although the marked advancement in CI technology over the past several years, Experiment 3′s findings suggest that it has yet to fully overcome the challenge of speech perception in noisy everyday environments.

## 5. General Discussion

Over the course of the three experiments of this study, we assessed the adaptive version of the HeBio test on young NH (Experiment 1), examined the effect of four age groups and two types of noise (Experiment 2), and evaluated the performance of prelingual CI users (Experiment 3). Presenting an adaptive version of the HeBio amidst four-talker babble noise evidenced normal threshold distribution among normal-hearing participants (a mean SRTn near 0 dB and a slope of 16%/dB) and high test–retest reliability for both sentence lists. The SRTn values were found to be higher than in other similar studies, but taking into account that the background noise used here was more variable and informational than in other studies, the results appear commensurate with those reported. Further, the Adaptive HeBio thresholds were sensitive to increases in age, showing decreased SRTn values from adolescent to young adult participants, and increased SRTn values with the increased ages from middle-aged to older adults, as expected from other tests and languages. The Adaptive HeBio was also sensitive to the type of noise, evidencing better thresholds (more negative) for energetic noise (SSN) and worse for informational noise (4TB). The results also showed that the Adaptive HeBio test is applicable to CI users, although high variability between subjects was demonstrated. Finally, the associations between performance on the Adaptive HeBio and perceiving CVC words in quiet, cognitive digit span test performance, and age at implantation, strengthen the relations between speech perception in noise and these variables that were found in previous studies that also tested CI users. A limitation of the adaptive version of the HeBio may be the fact that its sentences are not equal in intelligibility since each list includes low- and high-accuracy sentences [29,30]. However, this is mitigated by the fact that the lists were organized with the same method, resulting in both high and low intelligibility sentences in each list. This enables an interchangeable use of different lists as they will elicit similar thresholds. Indeed, the results showed no difference in SRTn between lists, and a similar threshold variability and range to other tests (Matrix in Hebrew [26]; HINT in English [49]). The similar SRTn and slopes demonstrated between the test lists using the adaptive procedure in Experiments 1 and 2 indicate that when evaluating NH at different age groups with the Adaptive HeBio test, there is no learning effect, and the use of one list may be sufficient. Another limitation of this test, as well as other speech-in-noise tests, is that it is suitable for those who receive more than 40% in sentences in quiet conditions.

In summary, we recommend using the Adaptive HeBio with all its 33 lists for NH and CI users. It has the advantages of an adaptive procedure in both clinical and research settings, having a relatively short testing time and minimal ceiling and floor effects, and a large number of lists, which allows the opportunity for testing in multiple conditions or repeated testing. Lower SRTn were obtained when HeBio was accompanied by SSN. We, therefore, recommend using it when testing CI users. Future studies should examine this adaptive test among postlingually adults with CI as well as among older adults with hearing aids.

## Figures and Tables

**Table 1:** Demographic and background data for the CI group.

Participant	Age (y)	Gender	Etiology	Type of CI	N. of CIs	Age at 1st CI	Age at 2nd CI
1	25.5	F	CMV	C	2	4	14
2	23	F	Genetic	C	2	2.5	13
3	23	F	Genetic	C	2	2.5	14
4	28	M	Unknown	C	1	6	-
5	30	F	Genetic	C	2	3	14
6	27	M	Genetic	C	2	3	26
7	28.5	F	Waardenburg	AB	1	8	-
8	25	M	Genetic	C	1	20	-
9	16	F	Unknown	C	2	1	10
10	16	M	Unknown	C	2	1	6
11	11	F	Unknown	C	2	1	4
12	26	M	Unknown	C	2	4	25
13	30	F	Genetic	C	2	3	14
14	20	F	Unknown	C	2	3	7
15	23	F	Unknown	C	2	2	11
16	19	M	Genetic	C	2	1	10
17	31	F	CMV	C	2	12	21
18	15	F	Genetic	C	2	4	4

(y) = years; Gender: F = female, M = male; CMV = Cytomegalovirus; Type of CI: C = Cochlear, AB = Advanced Bionics.

## Data Availability

The data presented in this study are openly available at: https://www.kaggle.com/datasets/leahfostick/adaptive-hebio (accessed on 28 June 2022).
